# Chronic Nocturnal Abdominal Pain as the Presentation of Inverted Meckel Diverticulum: A Case Report

**DOI:** 10.3390/children9010069

**Published:** 2022-01-05

**Authors:** Ting-Yu Wang, Yu-Tsun Su, Po-Jui Ko, Yea-Ling Chen, Hsiang-Hung Shih, Ching-Chung Tsai

**Affiliations:** 1 Department of Pediatrics, E-Da Hospital, Kaohsiung City 824005, Taiwan; stopright@gmail.com (T.-Y.W.); suyutsun@yahoo.com.tw (Y.-T.S.); amystella2001@yahoo.com.tw (Y.-L.C.); 2 Department of Pediatrics, U-Sheng Hospital, Pingtung City 900023, Taiwan; 3 School of Medicine, I-Shou University, Kaohsiung City 824005, Taiwan; 4 Department of Pediatric Surgery, E-Da Hospital, Kaohsiung City 824005, Taiwan; ed106790@edah.org.tw; 5 Department of Pediatrics, Kaohsiung Medical University Hospital, Kaohsiung City 807387, Taiwan; 1040561@gap.kmu.edu.tw

**Keywords:** inverted Meckel’s diverticulum, intussusception, nocturnal abdominal pain

## Abstract

The common clinical manifestations of Meckel’s diverticulum include painless lower gastrointestinal bleeding and intestinal obstruction due to intussusception. Intussusception induced by inverted Meckel’s diverticulum has rarely been reported; furthermore, there is no report thus far of chronic nocturnal abdominal pain as a presenting symptom in children with Meckel’s diverticulum. A 4-year-and-10-month-old girl with no significant history of previous illness presented with the sole complaint of chronic nocturnal abdominal pain for 3 months. The patient was reported to be asymptomatic during the day. A provisional diagnosis of chronic ileoileal intussusception was already under consideration in her previous hospital visits elsewhere. Physical examination revealed a soft, non-distended abdomen without tenderness. Imaging studies revealed ileoileal intussusception. Exploratory laparotomy showed ileoileal intussusception induced by an inverted Meckel’s diverticulum with ulceration. The patient underwent successful surgery and made a full recovery. We report this case to remind physicians that Meckel’s diverticulum should be considered in differential diagnosis of children presenting with the isolated symptom of chronic nocturnal abdominal pain.

## 1. Introduction

Meckel’s diverticulum is a remnant of the omphalomesenteric duct in the human embryo. There is a “rule of twos” to describe the features of Meckel’s diverticulum. It occurs in approximately 2% of the general population. A Meckel’s diverticulum is nearly 2 inches (5 cm) long, usually within 2 feet (approximately 60 cm) of the ileocecal valve and located on the antimesenteric border of the ileum. More than 60% of patients develop symptoms within 2 years of age. In addition, two types of ectopic mucosa can be seen in the diverticulum: gastric and pancreatic. Owing to its location in the right lower quadrant, symptoms are usually similar to those of acute appendicitis when inflamed [1,2].

Intussusception is a common abdominal emergency in children, especially in those younger than two years of age. The typical symptoms and signs of intussusception include intermittent abdominal pain, a palpable abdominal mass in the right upper quadrant, and “currant jelly stool.” The etiology of intussusception in children younger than two years of age is idiopathic; however, in those older than 2 years of age, probable lead points such as Meckel’s diverticulum, polyps, duplication cyst, or a tumor should be considered [3,4]. We report a case of inverted Meckel’s diverticulum presenting with atypical symptom of chronic nocturnal abdominal pain.

## 2. Case Presentation

A 4-year-and-10-month-old girl with no significant past history of illness presented with chronic nocturnal abdominal pain for 3 months. She always presented in a prone posture with elevated buttocks when having abdominal pain. There were no other associated symptoms such as fever, vomiting, diarrhea, purpura, melena, or rectal bleeding, and she was generally asymptomatic during the day. She was admitted to our hospital with symptoms and findings as described above. Her recent medical history is listed in Table 1.

**Figure 1:**
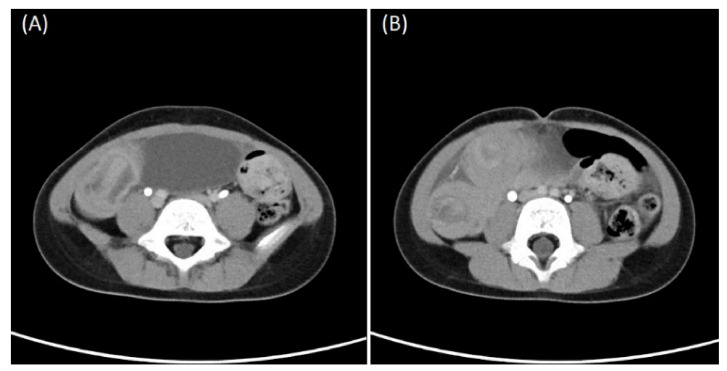
Computed tomography scan (axial view) revealing (**A**) cord-like structure inside and (**B**) ileoileal intussusception.

**Figure 2:**
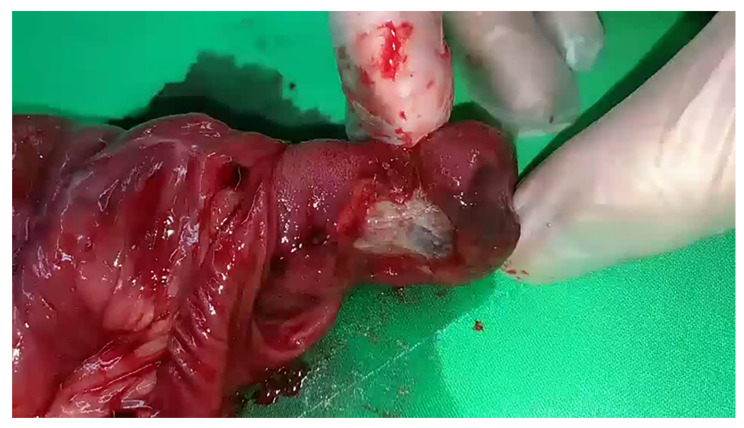
A 5-cm-long inverted cord-like structure with an ulceration is seen upon cutting the resected small bowel open.

**Figure 3:**
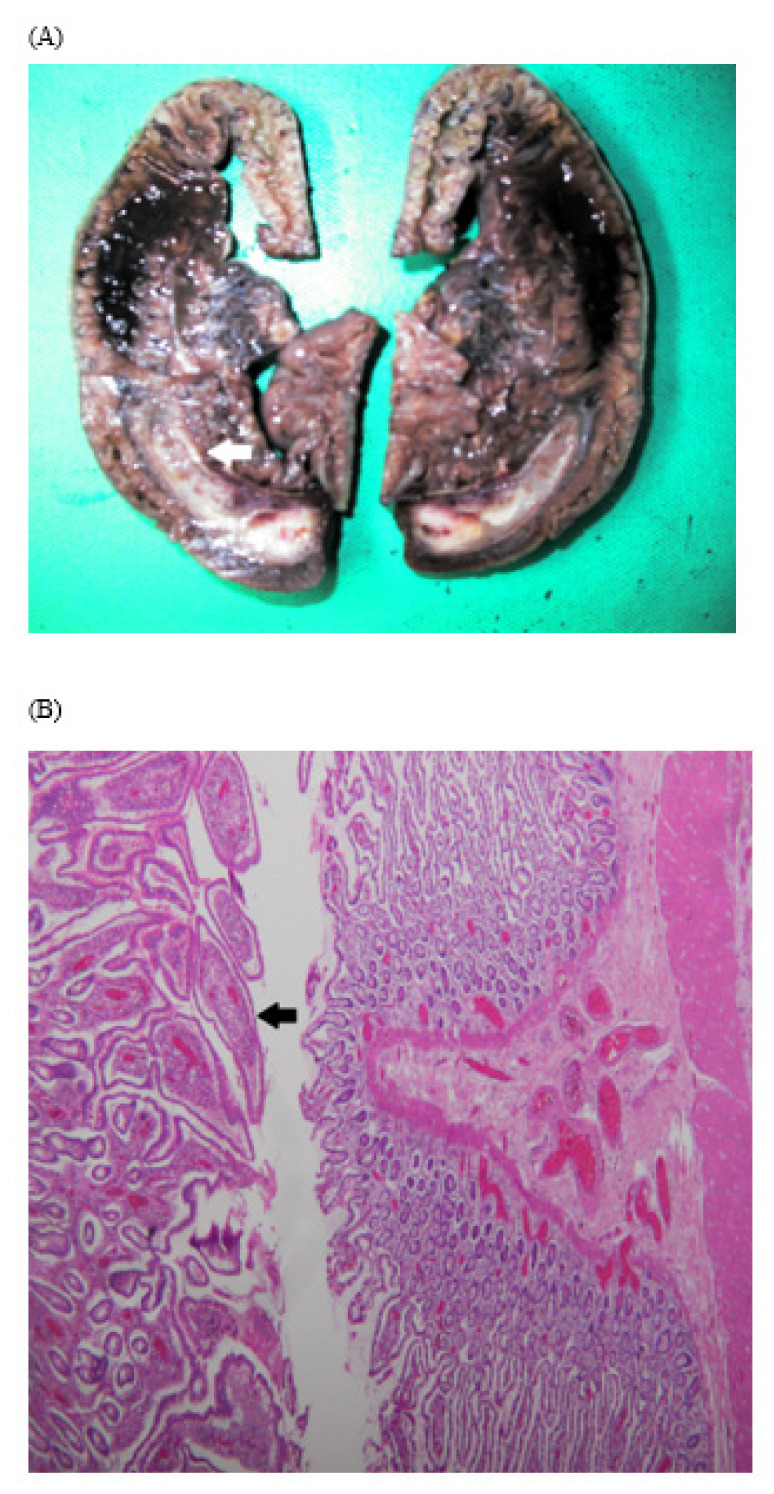
Pathology of resected bowel showing one segment of intestine measuring 15 cm in length. (**A**) One segment of rod-like structure 5 cm in length was observed inside (white arrow), and (**B**) the segment was lined by the small intestinal epithelium (black arrow).

Physical examination revealed a soft, non-distended abdomen without tenderness. Laboratory tests revealed WBC, 142,800/μL; neutrophils, 36.3%; eosinophils, 0.8%; lymphocytes, 56.5%; basophils, 1.6%; monocytes, 4.8%; erythrocyte sedimentation rate, 15 mm/h; aspartate aminotransferase, 22 U/L; alanine aminotransferase, 11 U/L; and creatinine, 0.7 mg/dL. The patient had two CTs (Figure 1), which revealed chronic intussusceptions requiring surgical exploration and exploratory laparotomy showed ileoileal intussusception with markedly dilated proximal small bowel loops without ischemic or necrotic changes (Video S1 in Supplementary Materials). The intussusception was carefully reduced, and a segment of the ileum with a mass was resected. Examination of the resected small bowel revealed a 5-cm-long inverted cord-like structure with ulcerations inside (Figure 2). The pathology report was consistent with an inverted Meckel’s diverticulum (Figure 3A,B). The postoperative recovery was smooth and uneventful.

## 3. Discussion

The exact mechanism of occurrence of inverted Meckel’s diverticulum is poorly understood due to the rarity of this condition. The probable causes of the inversion include abnormal peristalsis around the diverticulum, the diverticulum itself not being fixed, or its inversion due to abnormal peristalsis caused by ulcers or ectopic tissue at the bottom of Meckel’s diverticulum [5,6]. Therefore, based on these theories, the inverted Meckel’s diverticulum in this case may be secondary to the ulcer on Meckel’s diverticulum.

The common clinical manifestations of Meckel’s diverticulum include painless brick-red LGI bleeding, intestinal obstruction, diverticulitis, and perforation. In Meckel’s diverticulum, bleeding is caused by the secretion of acid from the ectopic gastric mucosa, which causes ulcers in the diverticulum or adjacent intestinal mucosa. Consequently, hypovolemic shock due to severe anemia secondary to painless massive bleeding can occur in many children, especially those younger than 2 years of age [7].

The probable reasons for nocturnal abdominal pain alone include the following: (1) chronic incompletely obstructing intussusception, (2) ulcerations on an inverted Meckel’s diverticulum without a buffer at night, and (3) it being quieter and easier to concentrate at night without the usual daytime distractions, which magnifies distress and seemingly increases a patient’s physical discomfort.

One common clinical manifestation of Meckel’s diverticulum is intestinal obstruction related to intussusception, especially in those older than 2 years of age [4]. In this case, successful reduction was achieved following the first medical treatment session, but chronic incompletely obstructing intussusception was suspected on attempted reduction during the second medical treatment session. Perhaps the bowel stiffness that was rendered by the inverted Meckel’s diverticulum gradually induced a dilated and looser lumen of the proximal small intestine via chronic intermittent incomplete intussusception. Hence, chronic non-strangulating incompletely obstructing intussusception was likely due to the looser and dilated proximal small intestine. This surmise was proven with observation of the looser and dilated lumen of the proximal small intestine during surgery in this case. Similar painless or painful chronic intussusception induced by lymphoma has also been reported [8,9]. Chronic incompletely obstructing, rather than acute and completely obstructing, intussusception may have been one of contributing factors of chronic intermittent nocturnal abdominal pain. The change in the prone posture and elevation of the buttocks could reduce chronic incompletely obstructing intussusception by the gravity of the intussuseptum.

There are many gastrointestinal diseases associated with abdominal pain at night, such as peptic ulcer, biliary disease, inflammatory bowel disease, irritable bowel syndrome, and gastroesophageal reflux disease (GERD) [10,11,12,13]. Nocturnal abdominal pain of peptic ulcer and GERD is associated with the secretion of acid in the empty stomach without any buffer. In addition, peptic ulceration of Meckel’s diverticulum or adjacent ileum has been rarely reported to cause pain along with bleeding [14]. In this case, ulcerations on the inverted Meckel’s diverticulum without a buffer at night may have been another contributing factor to the chronic intermittent nocturnal abdominal pain.

Clinically, intussusception caused by an inverted Meckel’s diverticulum or Meckel’s diverticulum in children manifesting as chronic nocturnal abdominal pain alone has rarely been reported. Therefore, we report a case of inverted Meckel’s diverticulum presenting with this atypical manifestation instead of typical painless LGI bleeding or intestinal obstruction.

## 4. Conclusions

In conclusion, we report this rare case with atypical clinical manifestations to remind physicians that Meckel’s diverticulum should be considered in children presenting with chronic nocturnal abdominal pain alone.

## Figures and Tables

**Table 1:** Summary of recent medical history prior to this admission.

Date	Hospital	Event
Seven months prior to this admission	A regional hospital	Intussusception was suspected on abdominal sonography, and it was resolved by lower gastrointestinal (LGI) series reduction
Three months prior to this admission	The same regional hospital as the 1st episode	Sonography suspected intussusception but LGI series revealed no evidence of intussusception. Incompletely obstructing intussusception was suspected.
One month prior to this admission	A medical center	Computed tomography (CT) suspected intussusception but upper gastrointestinal series showed negative finding. Incompletely obstructing intussusception was suspected again.

## Data Availability

Not applicable.

## Supplementary Materials

The following supporting information can be downloaded at: https://www.mdpi.com/article/10.3390/children9010069/s1, Video S1: Intussusception induced by inverted Meckel’s diverticulum and manual reduction.
